# Dopamine Response to Unexpected Aversive Outcomes Drives the Return of Extinguished Fear

**DOI:** 10.3390/brainsci16070690

**Published:** 2026-06-30

**Authors:** Bhumiben P. Patel, Jennifer Tat, Oyku Dinckol, Noah Harris Wenger, Aryanna Copling, Munir Gunes Kutlu

**Affiliations:** 1 Center for Substance Abuse Research, Temple University Lewis Katz School of Medicine, University in Philadelphia, Philadelphia, PA 19140, USA; bhumiben.patel@temple.edu (B.P.P.); oyku.dinckol.perni@temple.edu (O.D.); noah.wenger@temple.edu (N.H.W.); aryanna.copling@temple.edu (A.C.); 2 University of Tennessee Health Science Center, Memphis, TN 38163, USA; jenntat07@gmail.com; 3 Biomedical Sciences Graduate Program, Temple University Lewis Katz School of Medicine, University in Philadelphia, Philadelphia, PA 19140, USA; 4 Department of Neural Sciences, Temple University Lewis Katz School of Medicine, University in Philadelphia, Philadelphia, PA 19140, USA

**Keywords:** dopamine, fear extinction, reinstatement, nucleus accumbens

## Abstract

**Highlights:**

**What are the main findings?**

- Unexpected footshock prevented the decline of NAc core dopamine levels and reinstated extinguished fear.
- Enhancing dopamine signaling increased reinstatement, while inhibiting dopamine reduced fear relapse.

**What are the implications of the main findings?**

- NAc core dopamine signaling regulates the return of extinguished fear following unexpected aversive events.
- Dopamine circuits may represent a potential target for preventing the return of maladaptive threat responses.

**Abstract:**

**Background/Objectives**: Dopamine is well known for its role in reward learning, where phasic activity encodes prediction errors and supports the formation of cue–outcome associations. More recently, dopamine has been implicated in encoding salience, novelty, and aversive events, suggesting a broader function in shaping learning and memory across motivational contexts. However, how accumbal dopamine contributes to the recovery of extinguished fear, a process central to relapse in anxiety and trauma-related disorders, remains unclear. **Methods**: Here, we examined NAc core dopamine dynamics during shock-induced reinstatement of an extinguished fear memory. Using dLight fiber photometry, we found that a sustained decrease in baseline dopamine marked extinction recall. In contrast, an unexpected reminder footshock prevented this reduction, maintaining dopamine levels near baseline as freezing behavior re-emerged. **Results**: The reminder shock also evoked a transient dopamine peak, and optogenetic manipulations demonstrated that dopamine signaling during this period bidirectionally modulated reinstatement, with enhancement of dopamine release increasing reinstatement and inhibition of dopamine terminals markedly attenuating it. Together, these results demonstrate that unexpected aversive events reset NAc core dopamine levels and gate the return of extinguished fear. **Conclusions**: By revealing that accumbal dopamine contributes to fear recovery, this work broadens current models of dopamine function and identifies a neural mechanism through which surprising events may promote relapse of aversive memories in anxiety- and stress-related disorders.

## 1. Introduction

Dopamine has been extensively studied for its critical role in reward learning [1,2,3,4]. Phasic dopamine signals are robustly engaged by rewarding stimuli and their predictors, tracking a prediction error-based signal and supporting the encoding of predictive cue–outcome associations [5,6,7,8]. Moreover, emerging evidence suggests that dopamine is modulated by the saliency and novelty of external events and stimuli [9,10,11,12,13,14], dynamically integrating this information over time to signal environmental change and uncertainty [15], suggesting a broader role in shaping learning and memory beyond the classical reward prediction error framework. Beyond reward learning, these processes are increasingly recognized as important for adaptive responses to aversive experiences, and disruption in them contributes to maladaptive learning observed in psychiatric conditions such as substance use disorders, post-traumatic stress disorder (PTSD), and other anxiety or stress disorders [16,17]. Consistent with this, fear extinction and recovery depend on coordinated activity across the amygdala, medial prefrontal cortex (mPFC), hippocampus, and nucleus accumbens (NAc), with the NAc increasingly recognized as an integrative hub for motivational and salience-related signals that influence fear recovery [11,17,18,19,20].

Recent work has highlighted that dopamine is not limited to reward contexts but is also critically engaged during aversive learning. For example, dopamine neurons in the midbrain and their terminals in the nucleus accumbens (NAc) respond robustly to aversive stimuli and their predictors [11,12,21,22,23,24,25], and dopamine release is involved in active avoidance and conditioned punishment [11,26,27,28]. Studies from our group and others demonstrated that NAc core dopamine release is evoked by aversive stimuli, such as footshocks, as well as when those footshocks are predicted but absent [11,12,22,24,25,29]. Moreover, our group and others showed that this dopamine response during the unexpected omission of aversive outcomes causally regulates fear extinction and safety learning [11,18,25,29]. Specifically, we showed that optogenetic amplification of omission-induced dopamine release in the NAc core impaired fear extinction and safety learning. These findings suggest that dopamine may function as a teaching signal, signaling the unexpected presence or absence of important outcomes and thereby dynamically shaping how animals attend to, encode, and adapt their behavior to both threat- and safety-related experiences.

Previous work has shown that dopamine signaling contributes to both the acquisition and recall of extinction [11,18,27,30], and that dopamine transients during the omission of expected aversive outcomes promote fear extinction and safety learning [11,18,27,30]. However, its role in the re-emergence of fear after extinction remains unclear. Fear can return following extinction through several processes, including spontaneous recovery, contextual renewal, and reinstatement, which involve partially overlapping but distinct mechanisms [17,31]. Unlike spontaneous recovery or contextual renewal, reinstatement is triggered by renewed exposure to an aversive event following extinction, making it particularly relevant for understanding how unexpected threat experiences reactivate extinguished fear memories [31,32,33]. However, despite evidence implicating dopamine signaling in extinction learning and recall, the involvement of NAc core dopamine in the reinstatement of previously extinguished fear responses remains largely unknown. Therefore, in the present study, we specifically examined dopamine dynamics in the NAc core during shock-induced reinstatement of a previously extinguished fear memory. Clarifying this mechanism is essential for understanding how maladaptive fear responses re-emerge after extinction and may offer critical insight into the neural basis of relapse and recurrence in anxiety- and stress-related disorders.

## 2. Materials and Methods

Adult male and female C57BL/6J mice (6–8 weeks old; Jackson Laboratories, Bar Harbor, ME, USA; stock no. 000664) were housed five per cage and maintained on a 12-h reverse light/dark cycle with ad libitum access to food and water. All experimental procedures were approved by the Institutional Animal Care and Use Committee (IACUC) at Temple University and conducted in accordance with institutional guidelines.

### 2.1. Surgical Procedure

At least one hour before surgery, mice received ketoprofen (5 mg/kg, s.c.) for analgesia. Anesthesia was induced with 5% isoflurane and maintained at 2% while mice were secured in a stereotaxic frame (David Kopf Instruments, Tujunga, CA, USA). Using a 0.10-mL NanoFil syringe (WPI, Sarasota, FL, USA) fitted with a 34-gauge needle, AAV5.CAG.dLight1.1 (UC Irvine [34]) was unilaterally injected into the NAc (AP: +1.4 mm, ML: +1.5 mm, DV: −4.3 mm from bregma; 10° angle) at 50 nL/min for a total volume of 500 nL. The needle was left in place for 7 min before being slowly withdrawn. A fiber-optic cannula (400 μm core, 0.48 NA; Doric Lenses, Quebec, Canada) was then implanted immediately dorsal to the injection site and secured to the skull with adhesive cement (C&B Metabond). Mice were given at least 6 weeks to recover, allowing for robust viral expression prior to behavioral testing.

For optogenetic experiments, AAV5.Ef1a.DIO.hChR2.eYFP (ChR2; UNC vector core) or AAV5-Ef1a-DIO.eNpHR.3.0-eYFP (NpHR; Addgene, Watertown, MA, USA) and AAV9.rTH.PI.Cre.SV40 (Addgene) was injected into the VTA (unilaterally for ChR2 and bilaterally for NpHR; bregma coordinates: A/P: −3.16 mm; M/L: −0.5 mm; D/V: −4.8 mm; no angle) of C57BL/6J mice. Unilateral (for ChR2) or bilateral (for NpHR) 200 μm-core diameter fiber-optic implants were placed into the NAc core (bregma coordinates matching fiber photometry surgeries). This allowed for the photo-stimulation or photo-inhibition of dopamine responses only in dopamine axons projecting from the VTA and synapsing in the NAc core. Control animals received AAV5.Ef1a.DIO.eYFP injections into the VTA instead of ChR2 or NpHR. Bilateral optical inhibition was used to maximize suppression of dopaminergic signaling and reduce the potential for compensatory activity from the contralateral hemisphere, while unilateral stimulation was sufficient to reliably enhance dopamine signaling during reminder shock. We validated this optogenetic approach in our previously published studies [12,25].

### 2.2. Histology

Mice were deeply anesthetized with an intraperitoneal injection of ketamine/xylazine (100 mg/kg and 10 mg/kg, respectively) and transcardially perfused with 10 mL of 1× PBS followed by 10 mL of cold 4% paraformaldehyde (PFA) in PBS. Brains were removed, post-fixed in 4% PFA at 4 °C for at least 48 h and then transferred to 30% sucrose in PBS at 4 °C for cryoprotection. Once fully sunk, tissue was sectioned at 35 μm on a freezing sliding microtome (Leica SM2010R; Leica Biosystems, Deer Park, IL, USA) and stored in cryoprotectant solution (7.5% sucrose and 15% ethylene glycol in 0.1 M phosphate buffer) at −20 °C until processing. Sections were mounted onto glass slides with ProLong Gold (Invitrogen, Carlsbad, CA, USA) antifade reagent and imaged using a BZ-X700 inverted fluorescence microscope (Keyence, Itasca, IL, USA) equipped with a 20× dry objective (Nikon, Tokyo, Japan). Injection sites and optical fiber placements were verified from serial sections in all experimental animals (Supplementary Figure S2).

For the optogenetic experiments using AAV9.rTH.PI.Cre.SV40, we also validated the targeting of TH+ cells in the VTA via an anti-TH antibody (mouse anti-TH; Millipore #MAB318, 1:100). Sections were then incubated with secondary antibodies [GFP: goat anti-chicken AlexaFluor 488 (Life Technologies, Carlsbad, CA, USA #A-11039), 1:1000 and TH: donkey anti-mouse AlexaFluor 594 (Life Technologies # A-21203), 1:1000] for 2 h at room temperature. After washing, sections were incubated for 5 min with DAPI (NucBlue, Invitrogen, Carlsbad, CA, USA) to counterstain nuclei, then mounted in Prolong Gold. Representative images of viral expression and quantitative analysis of TH co-localization, and an anatomical map of VTA viral expression and NAc core fiber placements are provided in the Supplementary Figures S1–S4. Animals were prospectively evaluated using predefined exclusion criteria, including off-target viral expression or implant placement, poor optical signal quality, and technical issues during behavioral or neural recording. No animals met these exclusion criteria; therefore, no animals were excluded from the analyses.

### 2.3. Fiber Photometry

Fiber photometry recordings were obtained using a dual-LED system (490 nm and 405 nm; Thorlabs, Newton, NJ, USA) driven by an LED controller. The 490-nm LED, filtered through a 470-nm bandpass filter to match the excitation peak of dLight1.1, provided the dopamine-sensitive excitation, while the 405-nm LED served as an isosbestic control [34]. Light was delivered through a 400-µm, 0.48 NA optical fiber (Doric Lenses) coupled to the chronically implanted fiber-optic cannula in each mouse (fiber tip positioned above the NAc core). LED output was controlled by a real-time processor (RZ5P; Tucker-Davis Technologies, Alachua, FL, USA), and fluorescence emission was separated by multiplexing the two LED channels at distinct carrier frequencies, enabling demodulation of the 490-nm and 405-nm signals. Synapse software (Version 3) (Tucker-Davis Technologies, Alachua, FL, USA) regulated LED timing and intensity and acquired fluorescence signals detected by a photoreceiver (Newport Femtowatt Photoreceiver; Doric Lenses). LED power (125 µW) was measured daily at the fiber tip using a power meter (Thorlabs, Newton, NJ, USA) to ensure consistency across sessions. Behavioral events (e.g., cue onset or footshock delivery) were time-stamped using TTL pulses generated by Med-PC V (Med Associates, St. Albans, VT, USA) and recorded in Synapse via the RZ5P. A built-in 10-Hz low-pass filter in Synapse was applied to reduce high-frequency noise in the raw photometry signal prior to offline analysis. All recordings were performed in freely moving mice, and photometry signals were synchronized with behavioral data via TTL alignment.

### 2.4. Fiber Photometry Analysis

Raw F470 and F405 signals were first smoothed using locally weighted scatterplot smoothing (LOWESS; MATLAB, Natick, MA, USA “smooth” function, span = 0.0004). The smoothed F405 signal was then fitted to the smoothed F470 signal using a first-order polynomial regression across the full analyzed recording period. The fitted F405 signal was used to correct for motion-related artifacts and slow photobleaching-related signal drift. ΔF/F was calculated as (F470 − fitted F405)/fitted F405.

For event-based analyses, fluorescence traces were aligned to behavioral events using TTL timestamps, and data were extracted from −2 s to +17 s surrounding each event based on our previous published studies [11,12,15,25]. Z-scores were computed using the pre-event baseline period from −2 to 0 s for each event-aligned trace. Specifically, the mean and standard deviation of the −2 to 0 s baseline were calculated for each trial, and each time point in the corresponding peri-event trace was normalized as z = (signal − baseline mean)/baseline standard deviation. This approach enabled comparison of event-evoked dopamine responses across trials and animals.

For full-session trace analyses, ΔF/F signals were normalized using the same session-wide baseline derived from the early stable recording period (1–10 s after recording onset). The mean and standard deviation of this baseline window were used to z-score the entire trace.

To quantify dopamine responses, the area under the curve (AUC) was calculated from z-scored traces using trapezoidal numerical integration. Analysis windows were selected a priori based on the expected temporal dynamics of dopamine responses and previous fiber photometry studies. For baseline recordings, AUC was calculated across the entire recording period. For shock-evoked responses, AUC was calculated within a fixed post-shock time window that was applied consistently across all animals and experimental groups.

### 2.5. Behavioral Methods

**Figure 1:**
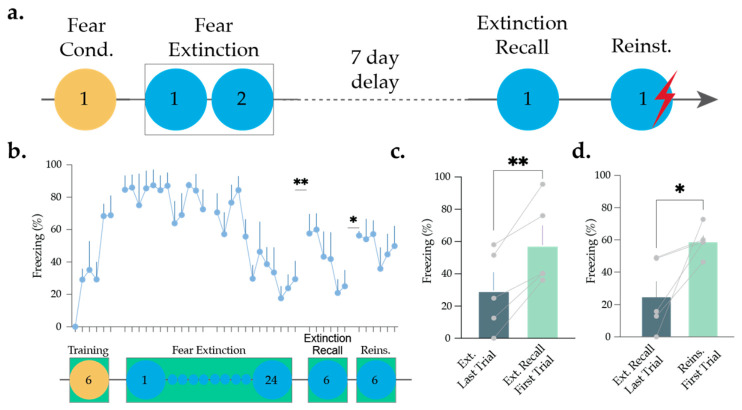
Unexpected footshock reinstates extinguished fear. (**a**) Mice (2 females and 3 males) underwent fear conditioning (Day 1), where an auditory cue (tone) was paired with an aversive footshock. This cue–footshock association was extinguished for two sessions (Days 2 and 3) by presenting the fear cue in the absence of the footshock outcome. After a 7-day hiatus, mice were tested in extinction for “spontaneous recovery” by presenting the extinguished cue alone for six trials. Finally, the following day, mice received six additional trials of the cue alone following a single reminder footshock to test for “reinstatement” of the extinguished cued fear response. (**b**) Mice showed fear conditioning (increase in freezing response during the training session) and fear extinction (gradual decrease in freezing during fear extinction; Repeated Measures ANOVA Trial main effect: *F*_(3.082,12.33)_ = 4.335, *p* = 0.0260, Sidak Post Hoc, Extinction Trial 1 vs. Trial 24, *p* < 0.01, *n* = 5 mice). (**c**) Following the 7-day delay, the freezing response showed a spontaneous recovery effect (Extinction Last Trial vs. Extinction Recall First Trial; paired *t*-test, *t*_4_ = 5.242, *p* = 0.0063, *n* = 5 mice). (**d**) Twenty-four hours later, the reminder shock resulted in the reinstatement of the extinguished freezing response to the fear cue (Extinction Recall Last Trial vs. Reinstatement First Trial; paired *t*-test, *t*_4_ = 3.328, *p* = 0.0292, *n* = 5 mice). Data represented as mean ± S.E.M. * *p* < 0.05, ** *p* < 0.01.

Mice were first trained in a fear conditioning paradigm (Figure 1a), where they received six pairings of tone (85 dB, 2.5-kHz frequency, 10 s in duration) and footshock (1 mA intensity and 0.5 s in duration; the onset of the footshock coincided with the tone offset) in a standard conditioning chamber (Med Associates) with distinct contextual cues (e.g., lighting, floor texture, and auditory cues) to facilitate context discrimination. Twenty-four hours after fear conditioning training, mice received 2 sessions of fear extinction, during which the tone was presented for 12 trials and footshocks were omitted (inter-trial interval: variable, ~60–120 s). Following a 7-day hiatus, mice underwent one additional extinction session (6 trials instead of 12) to assess extinction recall and spontaneous recovery. Cue-induced response and dopamine were measured throughout fear conditioning, extinction, and extinction recall sessions, with freezing behavior defined as the absence of all movement except respiration and manually scored by a trained observer blinded to experimental conditions. To induce reinstatement, mice were tested 24 h after the last fear extinction session and received a single unpaired footshock presentation (1 mA, 0.5 s) 30 s into the extinction recall session prior to presentation of the fear cue in extinction (no tone present during shock delivery). We recorded NAc mice via fiber photometry, as explained above, during the extinction recall and reinstatement test sessions, with behavioral events time-locked to photometry recordings via TTL signals to ensure precise alignment between neural and behavioral data. Unless stated otherwise, all behavioral and fiber photometry data were obtained from the same cohort of mice. Sample size was based on prior published studies utilizing similar behavioral, fiber photometry, and optogenetic approaches in fear-learning paradigms [11,12,15,25]. Both male and female mice were included in all experiments and analyzed together. Since the study was not powered to test sex differences, sex-specific analyses were not performed.

### 2.6. Optogenetic Inhibition/Stimulation of the NAc Core Dopamine Release at the Time of the Reinstatement Footshock

Three groups of mice were trained in fear conditioning and fear extinction and tested in extinction recall (24 h after the extinction session instead of 7 days) and reinstatement as described above, using identical behavioral parameters and contexts as described for the primary experiments. The mice that were injected with the excitatory opsin (channelrhodopsin, ChR2) and eYFP-expressing control mice received a blue laser (470 nm, 1 s, 20 Hz, 8 mW) during the single presentation of the footshock during reinstatement, whereas the group that received the inhibitory opsin halorhodopsin (NpHR) received yellow laser stimulation (590 nm, 1 s, constant, 8 mW) delivered through implanted optical fibers targeting the NAc core. Laser onset was precisely time-locked to the onset of the footshock via TTL-triggered stimulation, ensuring temporal specificity of the manipulation. The freezing response to cue presentation following the reminder shock was measured, with freezing defined as the absence of all movement except respiration, and was manually scored by observers blinded to experimental conditions. Light power was measured at the fiber tip prior to each session to ensure consistent light delivery across animals.

### 2.7. Statistical Analysis

Data were analyzed using Prism (version 11; GraphPad) and are presented as mean ± S.E.M. Sample Size (*n* = 5–7 animals/group) was selected based on prior published studies utilizing similar behavioral, fiber photometry, and optogenetic approaches in fear-learning paradigms [11,12,15,25]. Normality was assessed for all datasets using the Kolmogorov–Smirnov test. All groups passed normality testing (*p* > 0.1000), except for the normalized freezing responses for the optogenetics experiments, for which the normality assumption was not met, and a Mann–Whitney U test (normality not assumed) was used. Statistical comparisons were performed using Repeated Measures or two-way ANOVA, as appropriate, when assumptions of normality were met, followed by Sidak’s or Dunnett’s post hoc multiple-comparison tests.

## 3. Results

### 3.1. An Unexpected Footshock Increases NAc Core Dopamine Baseline and Reinstates Previously Extinguished Fear Response

Mice were first trained using a fear conditioning paradigm consisting of six tone–footshock pairings, which produced a robust increase in cue-evoked freezing across trials, followed by repeated extinction sessions (two sessions, Days 2–3), during which presentation of the tone in the absence of shock resulted in a reduction in freezing behavior across extinction sessions (Figure 1b; one-way ANOVA Trial main effect: *F*_(3.082,12.33)_ = 4.335, *p* = 0.0260, Sidak Post Hoc, Extinction Trial 1 vs. Trial 24, *p* < 0.01, *n* = 5 mice). After a 7-day hiatus, mice were tested for extinction recall, during which freezing response to the cue was recovered initially and decreased again at the end of the test session, consistent with spontaneous recovery due to the delay after the extinction session (Figure 1b,c; Extinction Last Trial vs. Extinction Recall First Trial; paired *t*-test, *t*_4_ = 5.242, *p* = 0.0063, *n* = 5 mice). On the following day, a single unpaired reminder footshock was delivered prior to tone presentation to induce reinstatement. The reminder footshock produced a clear reinstatement effect, with freezing to the cue re-emerging during the reinstatement test (Figure 1b,d; Extinction Recall Last Trial vs. Reinstatement First Trial; paired *t*-test, *t*_4_ = 3.328, *p* = 0.0292, *n* = 5 mice) to levels significantly above extinction recall (paired *t*-test, *t*_4_ = 2.946, *p* = 0.0421), indicating recovery of the conditioned fear response.

**Figure 2:**
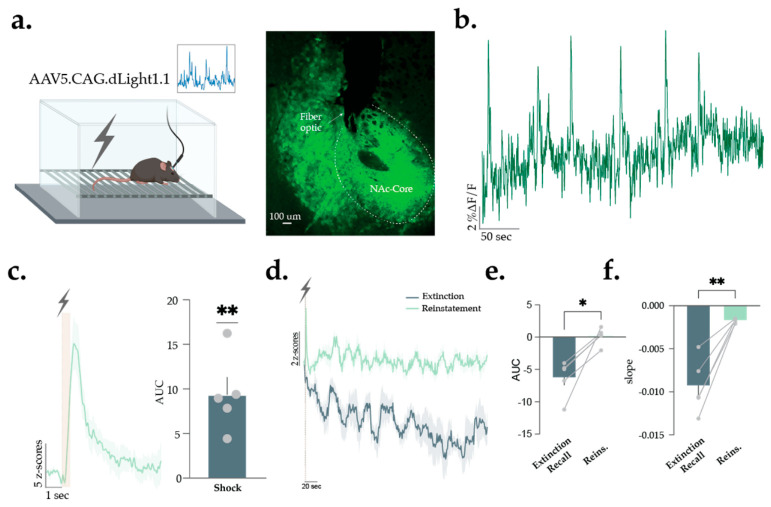
Reminder footshock prevents NAc core dopamine baseline decline during fear recovery. (**a**) Viral expression and fiber placement in the NAc core. Schematic of unilateral AAV5.CAG.dLight1.1 injection and optical fiber implantation targeting the nucleus accumbens (NAc) core (left). Representative histological image showing robust dLight1.1 expression and the location of the implanted fiber optic cannula within the NAc core (right). (**b**) Example fiber photometry signals from the NAc core. The representative processed ΔF/F signal demonstrates dopamine-dependent fluorescence fluctuations used for analysis. (**c**) The reminder footshock evoked a robust dopamine release in the NAC core (one-sample *t*-test, *t*_4_ = 4.866, *p* = 0.0082, *n* = 5 mice). (**d**) NAc core dopamine response during the extinction test (spontaneous recovery) decreased below baseline, whereas following the reinstating reminder, footshock remained at baseline. (**e**) The area under the curve (AUC) of the dopamine release at the NAc core terminals was significantly higher during reinstatement compared to the spontaneous recovery session (paired *t*-test, *t*_4_ = 4.127, *p* = 0.0145, *n* = 5 mice). (**f**) The slope of the dopamine baseline decrease was significantly larger during the spontaneous recovery session compared to dopamine during reinstatement, where the slope of the dopamine curve was around zero (paired *t*-test, *t*_4_ = 5.597, *p* = 0.0050, *n* = 5 mice). Data represented as mean ± S.E.M. * *p* < 0.05, ** *p* < 0.01.

NAc core dopamine release was continuously recorded during both extinction recall and reinstatement phases using dLight fiber photometry in order to test how dopamine activity relates to the return of freezing behavior following a reminder shock (Figure 2a,b; see Supplementary Figure S1 for the placement of the optogenetic implants). The reminder footshock itself evoked a robust dopamine response in the NAc core (Figure 2c; one-sample *t*-test, *t*_4_ = 4.866, *p* = 0.0082), consistent with rapid dopamine signaling to salient aversive events. During extinction recall, NAc core dopamine exhibited a reduction below baseline over the course of the session (Figure 2d), whereas following reminder footshock, this decline was not observed during reinstatement, with dopamine levels remaining near baseline, indicating that the reminder shock stabilizes or elevates dopamine signaling during recall. Quantification of these dynamics revealed that dopamine signaling was significantly elevated during reinstatement compared to extinction recall (AUC; paired *t*-test, *t*_4_ = 4.127, *p* = 0.0145; Figure 2e), and that the slope of the signal showed a significant decline during extinction recall but remained near zero during reinstatement (paired *t*-test, *t*_4_ = 5.597, *p* = 0.0050; Figure 2f). Thus, unexpected aversive stimulation prevented the baseline reduction in dopamine release normally observed during recall.

These findings indicate that unexpected footshock alters NAc core dopamine dynamics in a manner that permits the return of an extinguished fear response, linking shock-evoked dopamine signaling and sustained changes in dopamine baseline to behavioral reinstatement, consistent with the broader sensitivity of accumbal dopamine to surprising events, including both novelty and unpredicted aversive stimuli, during fear recovery.

### 3.2. NAc Core Dopamine Signaling During Unexpected Footshocks Contributes to Reinstatement Following Extinction

**Figure 3:**
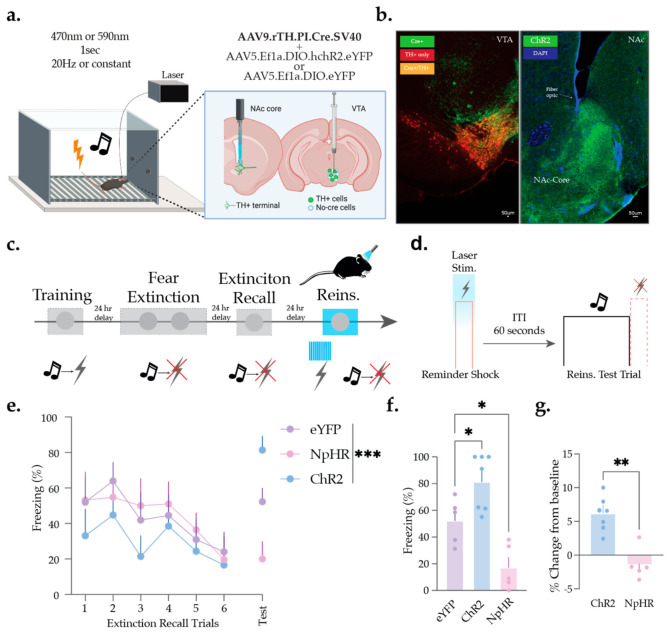
Stimulating dopamine response to the unexpected footshock enhances the reinstatement of extinguished fear. (**a**) Photoinhibition/stimulation was done on NAc core dopamine axons during reinstatement of previously extinguished freezing response to an auditory cue (tone). This was achieved via the expression of an excitatory (AAV5.Ef1a.DIO.hChR2.eYFP; ChR2) or an inhibitory opsin (AAV5-Ef1a-DIO.eNpHR.3.0-eYFP; NpHR) expressed in combination with a TH-specific Cre virus (AAV9.rTH.PI.Cre.SV40), all of which were injected directly into the VTA. Fiber optics was then placed above the NAc core, allowing for laser pulses to be delivered to the axons expressing each opsin in a dopamine- and projection-specific fashion. (**b**) Representative histology showing viral expression of channelrhodopsin (ChR2; green) in axons in the NAc core and viral expression of Cre-dependent channelrhodopsin (Cre+, green) restricted to the TH+ cells (red) in the VTA (Cre+/TH+ overlap, orange). (**c**) Mice went through fear conditioning (Training, Day 1) and fear extinction (Days 2–3), followed by an extinction recall (spontaneous recovery) and reinstatement test. (**d**) A photostimulating (blue, 470 nm, 1 s, 20 Hz)/inhibiting (yellow, 590 nm, 1 s, constant) laser was delivered at the time of the reminder footshock during the reinstatement test. (**e**,**f**) Following the presentation of the reminder footshock, ChR2 mice showed a stronger freezing response to the extinguished fear cue, while the NpHR mice showed a weaker freezing response compared to the eYFP controls (one-way ANOVA group main effect for the test day: *F*_(2,14)_ = 17.09, *p* = 0.0002; ChR2 vs. eYFP Control Dunnett’s post hoc *p* = 0.0342; NpHR vs. eYFP Control Dunnett’s post hoc *p* = 0.0191; *n* = 5–7 per group). (**g**) The ChR2 group (3 females and 4 males) also exhibited a larger shock-induced reinstatement effect (%increase compared to the last extinction recall cue response) than the NpHR group (2 females and 3 males) (Mann–Whitney U test, *p* = 0.0051; *n* = 5–7 per group). Data represented as mean ± S.E.M. * *p* < 0.05, ** *p* < 0.005, *** *p* < 0.001.

Having established that an unexpected footshock alters NAc core dopamine dynamics and coincides with the return of an extinguished fear response, we next asked whether these dopamine signals causally contribute to reinstatement. To address this, we manipulated NAc core dopamine release precisely at the time of the reminder footshock using temporally precise, TTL-triggered optogenetic control. Using optogenetics, we either inhibited dopamine terminals with halorhodopsin (NpHR) or stimulated them with channelrhodopsin (ChR2) during the reminder shock (Figure 3a,b), while eYFP-expressing mice served as controls for nonspecific effects of light delivery, and then assessed reinstatement during subsequent cue-alone presentations (Figure 3c,d).

Compared to eYFP controls, ChR2 stimulation enhanced reinstatement, producing a markedly stronger return of freezing to the cue (Figure 3e–g) relative to extinction recall levels. In contrast, NpHR-mediated inhibition sharply reduced reinstatement, with mice showing little to no recovery of the fear response (Figure 3e–g) despite receiving the same reminder shock (one-way ANOVA, main effect of group for the test day: *F*_(2,14)_ = 17.09, *p* = 0.0002; ChR2 vs. eYFP: *p* = 0.0342; NpHR vs. eYFP: *p* = 0.0191). These bidirectional effects indicate that the dopamine surge evoked by the unexpected footshock contributes to reinstatement and, when amplified, can potentiate the recovery of extinguished fear in a temporally specific manner tied to the aversive event.

Together with our photometry findings, these results identify a functional mechanism in which unexpected events, whether aversive or novel, reset NAc core dopamine levels, thereby shaping how external cues regain behavioral control following extinction by gating the transition from suppressed to reinstated fear states.

## 4. Discussion

Here, we examined how dopamine release in the NAc core shapes the reinstatement of an extinguished fear memory. During extinction recall, NAc core dopamine levels declined below baseline, whereas an unexpected reminder shock prevented this decline, maintaining dopamine levels near baseline and coinciding with renewed freezing. Optogenetic manipulations further supported a role for dopamine signaling in reinstatement: stimulation of dopamine axons at the time of the reminder shock amplified reinstatement, whereas inhibition markedly blunted it. These results identify dopamine release during unexpected aversive events as a critical regulator of fear relapse.

Recent findings from our group have demonstrated that novelty and unexpected sensory changes in the environment, such as an unexpected presentation of a neutral tone, produce robust increases in NAc core dopamine release [15]. Importantly, this elevation in dopamine baseline persisted well beyond the time window for the presentation of the novel stimulus and altered how familiar cues were subsequently processed. The present results build directly on this framework. Our results suggest that the sustained dopamine reduction during extinction recall may reflect the encoding of reduced saliency of the previously learned aversive state, consistent with prior observations of dopamine decreases during low-threat conditions [11,35,36]. Importantly, an unexpected aversive event prevented the reduction in dopamine observed during extinction recall, thereby enabling the re-expression of a previously extinguished fear response. Taken together, these studies suggest that NAc core dopamine serves as a general detector of unexpected environmental change, whether neutral, novel, or aversive, and that such signals can reorganize downstream processing in ways that either facilitate learning about new information or reinstate dormant threat associations. Although changes in behavioral state, including freezing-related immobility, may influence dopamine signaling during extinction recall, the prevention of dopamine decline following a reminder shock despite renewed freezing suggests that freezing-related immobility is unlikely to fully account for the observed dopamine responses. Future studies will incorporate detailed movement tracking, which will help further dissociate behavioral state from dopamine signaling during fear recovery.

A prediction-error framework also offers an interpretation of these findings. In classical models, unexpected outcomes, whether better or worse than predicted, evoke phasic changes in dopamine that update internal representations of the environment [1,6]. In our paradigm, the reminder footshock occurred after a period in which the animal had learned that the cue no longer predicted shock, making it an unexpected aversive event that may engage processes consistent with an aversive prediction error, heightened salience, or novelty. Such a violation of expectation may contribute to the transient increase in dopamine, consistent with the photometry signal observed at the time of the reminder shock. The dopamine response could then promote renewed engagement of the previously extinguished association and reinstatement of freezing. From this perspective, the dopamine surge at the moment of an unexpected shock may function as a teaching or updating signal that promotes re-expression of the threat memory, while optogenetic enhancement or suppression of this signal modulates the strength of reinstatement.

Importantly, dopamine responses to aversive events are heterogeneous across mesolimbic circuits, with distinct VTA dopamine subpopulations and NAc subregions showing differential responses to aversive stimuli and fear-related learning [11,12,21,22,24]. Thus, our findings specifically implicate NAc core dopamine in reinstatement and may not be interpreted as a uniform dopaminergic response across the mesolimbic circuitry. Nevertheless, these findings suggest that dopamine also gates the re-expression of previously suppressed aversive memories. Traditional frameworks emphasize dopamine’s roles in prediction error, salience detection, and safety learning [1,6,11,12,25,37], but the present findings indicate that dopamine may also influence whether extinguished threat associations remain suppressed or become reactivated following unexpected environmental changes. This expands dopamine’s influence from shaping how new associations are acquired to regulating whether old associations are permitted to re-emerge. Alongside our results showing that novelty increases NAc dopamine and transforms stimulus processing, our findings suggest that dopaminergic responses to unexpected change, whether innocuous or aversive, serve as a shared pathway through which such events restore behavioral control and contribute to the persistence and recurrence of maladaptive fear.

Although caution should be exercised when extrapolating findings from rodent fear reinstatement models to human psychopathology, the present results may provide insight into neural mechanisms that contribute to the recovery of extinguished fear. Fear reinstatement is commonly used as a preclinical model for studying the return of fear following successful extinction and may capture certain features relevant to trauma- and anxiety-related disorders. In this context, our findings suggest that dopaminergic responses to unexpected aversive events may contribute to the re-emergence of previously extinguished fear memories. Specifically, unexpected aversive experiences may engage nucleus accumbens dopamine signaling in a manner that promotes the recovery of latent threat associations. Consistent with our previous work demonstrating that accumbal dopamine neurons are sensitive to salient, surprising, and novel events [11,12,15,25], the present findings further support a role for dopamine signaling in updating behavioral responses when environmental contingencies change. Future studies will be needed to determine whether similar mechanisms contribute to maladaptive fear recovery in clinical populations and how dopamine signaling interacts with other neural systems implicated in trauma- and anxiety-related disorders.

Despite these findings, a few limitations should be considered. Although sample sizes were consistent with prior studies, the modest group sizes may limit the generalizability of the findings. Moreover, while the reminder shock prevented the decline in dopamine observed during extinction recall and bidirectionally altered reinstatement behavior, supporting a contribution of dopamine signaling to fear recovery. However, because reinstatement was examined only after reminder shock, the extent to which these effects reflect shock-induced reinstatement rather than spontaneous recovery or contextual exposure remains unclear. Future studies incorporating a no-shock control group will be important for addressing this distinction. In addition, while our optogenetic findings are consistent with a role for dopamine signaling, a subset of VTA TH+ neurons co-release glutamate at accumbal terminals, raising the possibility that co-transmission contributed to the observed behavioral effects. Additionally, because the eYFP control group received blue light stimulation, whereas the NpHR group received yellow light, nonspecific effects related to wavelength or optical stimulation parameters cannot be fully excluded. Future studies incorporating wavelength-matched controls, greater cell-type specificity, and temporally controlled manipulations outside the reminder shock window will help determine whether dopamine activity during the shock specifically contributes to reinstatement or reflects a broader mechanism through which unexpected events reactivate previously extinguished fear responses.

Together, these findings show that NAc core dopamine is a key factor in restoring the expression of extinguished threat memories in response to unexpected events. Unpredicted aversive stimuli elevate dopamine, prevent recall-associated decline, and contribute to reinstatement, supporting a mechanism for dormant fear responses’ re-emergence. Alongside recent work showing that novelty also elevates dopamine and reshapes processing, this study demonstrates that dopamine is a signal for unexpected change that governs whether past aversive memories remain suppressed or regain control over behavior. This framework clarifies the mechanistic basis for understanding fear relapse and highlights dopaminergic circuits as a potential target for preventing the return of maladaptive threat.

## 5. Conclusions

In conclusion, here we show that unexpected aversive events restore the expression of extinguished fear by engaging dopamine signaling in the NAc core. Reminder footshocks prevented the decline in dopamine observed during extinction recall, and optogenetic manipulations demonstrated that dopamine signaling at the time of the aversive event bidirectionally controls fear reinstatement. These findings identify NAc core dopamine as a critical regulator of fear recovery and support a broader role for dopamine in signaling unexpected environmental change that can reactivate previously suppressed threat memories.

## Data Availability

The data supporting the findings of this study are available within the article and its Supplementary Materials. Additional information may be obtained from the corresponding authors.

## Supplementary Materials

The following supporting information can be downloaded at: https://www.mdpi.com/article/10.3390/brainsci16070690/s1, Figure S1: Schematic showing the approximate location of fiber optic placements in both ChR2 and NpHR (red dots) as well as dLight (green dots) animals. Figure S2: Representative histology showing viral expression of halorhodopsin (eNpHR3.0; green) in axons within the NAc core and viral expression of Cre-dependent halorhodopsin (Cre+, green) restricted to TH+ neurons (red) in the VTA (Cre+/TH+ overlap, orange). An optical fiber was implanted above the NAc core to permit optogenetic inhibition of VTA dopamine terminals. Figure S3: Quantification of viral targeting specificity in the VTA. Across all animals (n = 12; ChR2 n = 7, NpHR n = 5), ~75% of Cre+ cells co-localized with TH immunoreactivity, indicating preferential opsin expression in dopaminergic neurons. Co-localization was significantly greater than the 50% reference value (one-sample *t*-test, t11 = 3.919, *p* = 0.0024). Figure S4: Reconstruction of viral expression within the VTA for all animals included in the optogenetic experiments. Coronal atlas sections show the extent of ChR2 (green; left) and NpHR (yellow; right) expression across animals, with each shaded region representing viral spread from an individual subject. Overlapping regions indicate areas of consistent viral expression within the VTA across the cohort.

## Abbreviations

The following abbreviations are used in this manuscript:

PTSD	Post-traumatic stress disorder
NAc	Nucleus Accumbens
NpHR	Halorhodopsin
ChR2	Channelrhodopsin
AUC	Area under the curve
