# Supplementary material for: Dopamine Response to Unexpected Aversive Outcomes Drives the Return of Extinguished Fear
Source: Brain Sci. 2026 Jun 30;16(7):690. doi: 10.3390/brainsci16070690 (PMC13406584; doi:10.3390/brainsci16070690)
Supplement: Supplementary file 1 [file brainsci-16-00690-s001.zip › brainsci-4342303-supplementary.pdf]

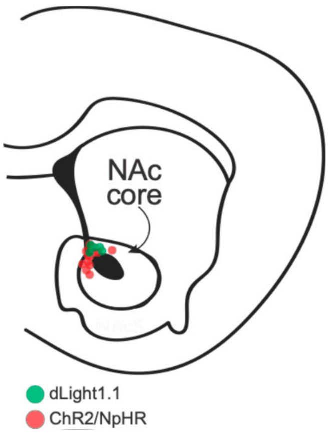

Figure S1. Schematic showing the approximate location of fiber optic placements in both ChR2 and NpHR (red dots) as well as dLight (green dots) animals.

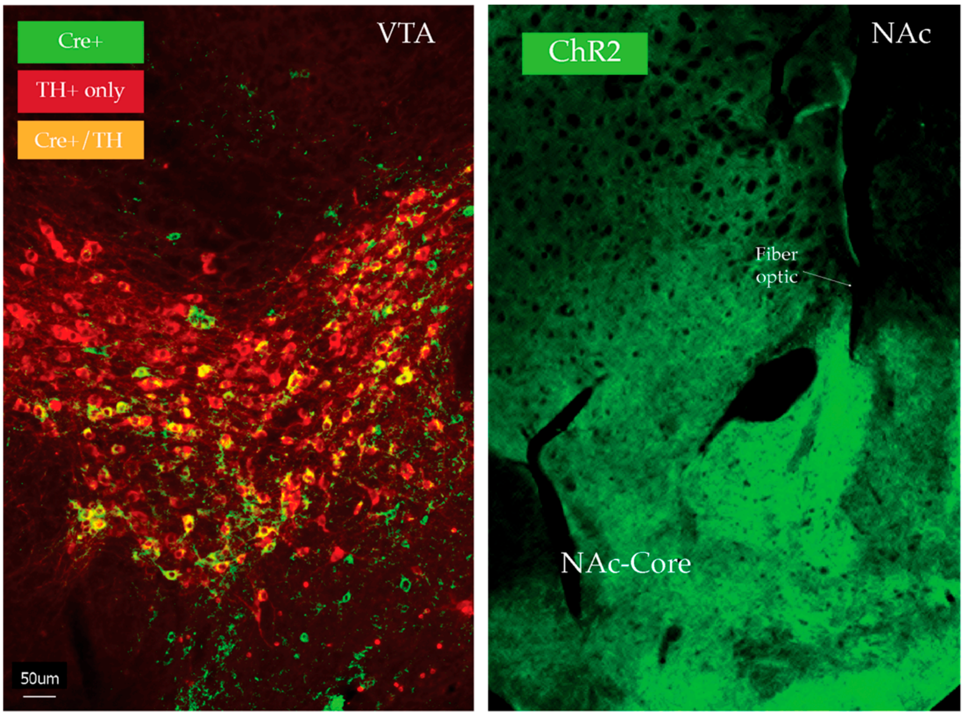

Figure S2. Representative histology showing viral expression of halorhodopsin (eNpHR3.0; green) in axons within the NAc core and viral expression of Cre-dependent halorhodopsin (Cre+, green) restricted to TH+ neurons (red) in the VTA (Cre+/TH+ overlap, orange). An optical fiber was implanted above the NAc core to permit optogenetic inhibition of VTA dopamine terminals.

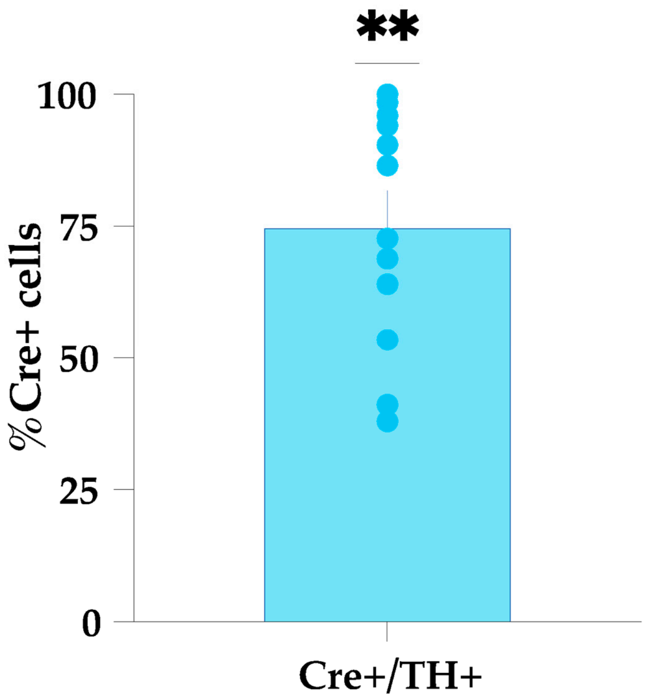

Figure S3. Quantification of viral targeting specificity in the VTA. Across all animals (n = 12; ChR2 n = 7, NpHR n = 5), ~75% of Cre+ cells co-localized with TH immunoreactivity, indicating preferential opsin expression in dopaminergic neurons. Co-localization was significantly greater than the 50% reference value (one-sample t-test, $t_{11} = 3.919$, $p = 0.0024$).

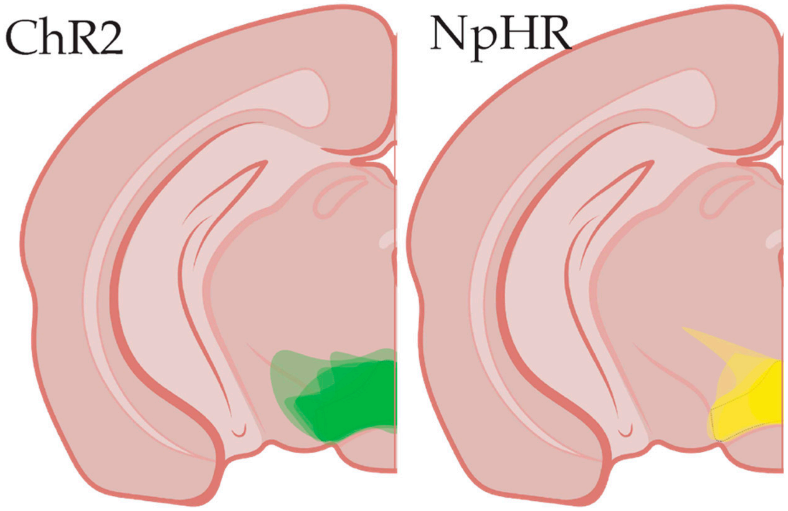

Figure S4. Reconstruction of viral expression within the VTA for all animals included in the optogenetic experiments. Coronal atlas sections show the extent of ChR2 (green; left) and NpHR (yellow; right) expression across animals, with each shaded region representing viral spread from an individual subject. Overlapping regions indicate areas of consistent viral expression within the VTA across the cohort.
